# Author Correction: Transcriptome analysis in a humanized mouse model of familial dysautonomia reveals tissue-specific gene expression disruption in the peripheral nervous system

**DOI:** 10.1038/s41598-024-60576-8

**Published:** 2024-05-02

**Authors:** Ricardo Harripaul, Elisabetta Morini, Monica Salani, Emily Logan, Emily Kirchner, Jessica Bolduc, Anil Chekuri, Benjamin Currall, Rachita Yadav, Serkan Erdin, Michael E. Talkowski, Dadi Gao, Susan Slaugenhaupt

**Affiliations:** 1 Center for Genomic Medicine, Massachusetts General Hospital Research Institute, Boston, MA USA; 2 Department of Neurology, Massachusetts General Hospital and Harvard Medical School, Boston, MA USA; 3 Program in Medical and Population Genetics and Stanley Center for Psychiatric Research, Broad Institute of Harvard and MIT, Cambridge, MA USA

Correction to: *Scientific Reports* 10.1038/s41598-023-51137-6, published online 04 January 2024

The original version of this Article contained an error in the Funding section.

“Research support from PTC Therapeutics, Inc. (S.A.S. and E.M.).”

now reads:

“NIH R37NS095640 to S.A.S. and HMS Ruth and Maurice Freeman Award for Pain Research to E.M.”

The original Article has been corrected.

