# Supplementary material for: Transcriptome analysis in a humanized mouse model of familial dysautonomia reveals tissue-specific gene expression disruption in the peripheral nervous system
Source: Sci Rep. 2024 Jan 4;14:570. doi: 10.1038/s41598-023-51137-6 (PMC10766950; doi:10.1038/s41598-023-51137-6)
Supplement: Supplementary file 1 — Supplementary Information. [file 41598_2023_51137_MOESM1_ESM.docx]

**Supplementary Table S1. Transcriptome-wide statistics of five tissues in the DEG analysis.**

**Supplementary Table S2. Full list of GO enrichment of DEGs from five tissues.**

**Supplementary Table S3. Tissue-specific co-expression modules that contain *Elp1*.**

**Supplementary Table S4. Full list of tissue-specific *ELP1* dose-responsive genes.**

**Supplementary Table S5. Connectivity scores of nodes in the tissue-specific FD-dysregulated gene networks according to StringDB.**

**Supplementary Table S6. Full list of GO enrichment for the FD signature genes involved in each tissue-specific FD-dysregulated gene network.**

**Supplementary Table S7. Full list of GO enrichment for the PNS-convergent FD signature genes.**

**Supplementary Table S8. Neuronal subtype markers identified from the re-analyses of public-available scRNA datasets.**


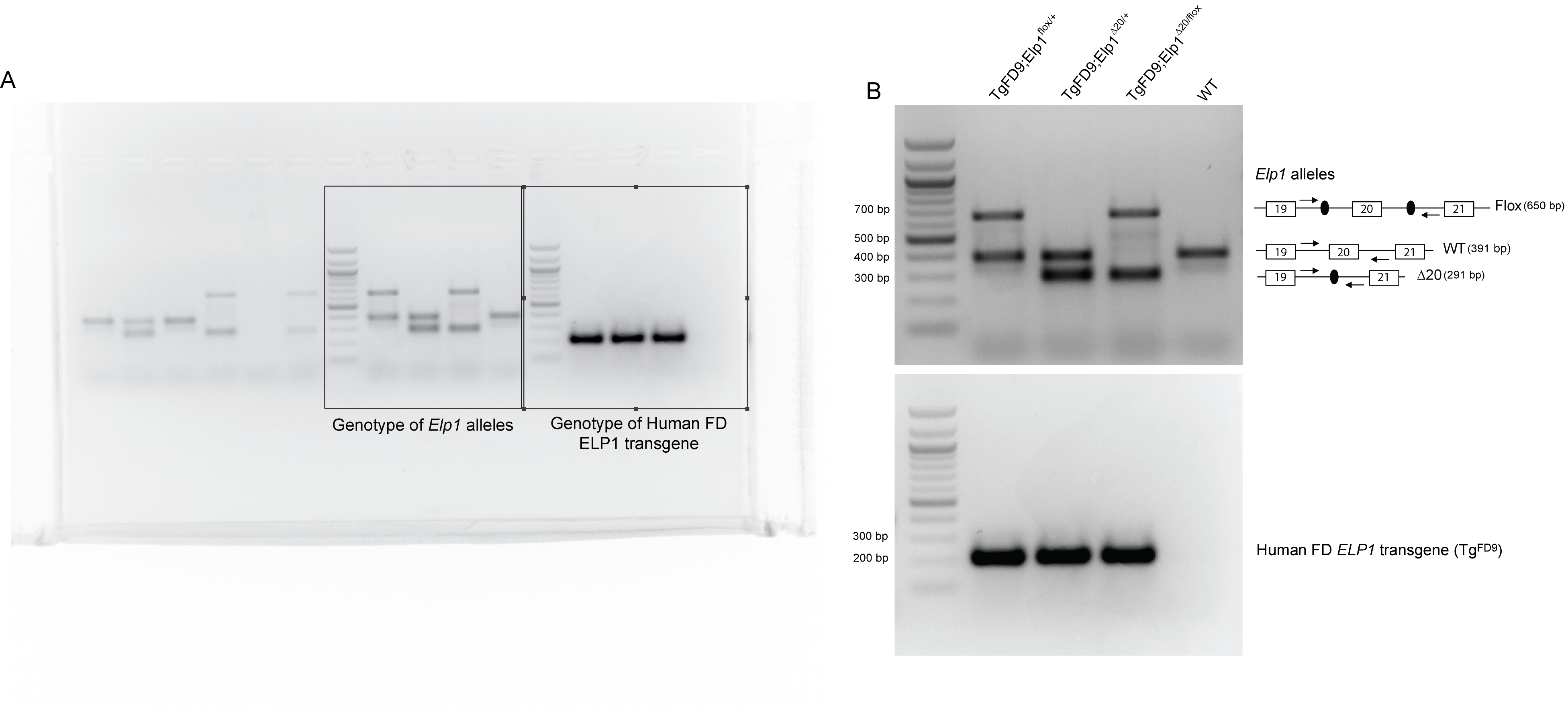


**Supplementary Figure S1. The humanized FD phenotypic mouse *TgFD9; Elp1^Δ20/flox^*.**

A) Original image of the gel used to generate the panels for this figure. B) Detection of *Elp1* alleles and human *TgFD9 ELP1* transgene in *TgFD9/Elp1^flox/+^* (control littermate), *TgFD9/Elp1^Δ20/+/+^* (control littermate)*,* *TgFD9/Elp1^Δ20/flox^* (FD phenotypic) and WT (control) mouse by PCR on genomic DNA. All PCR products were sequenced to confirm identity.


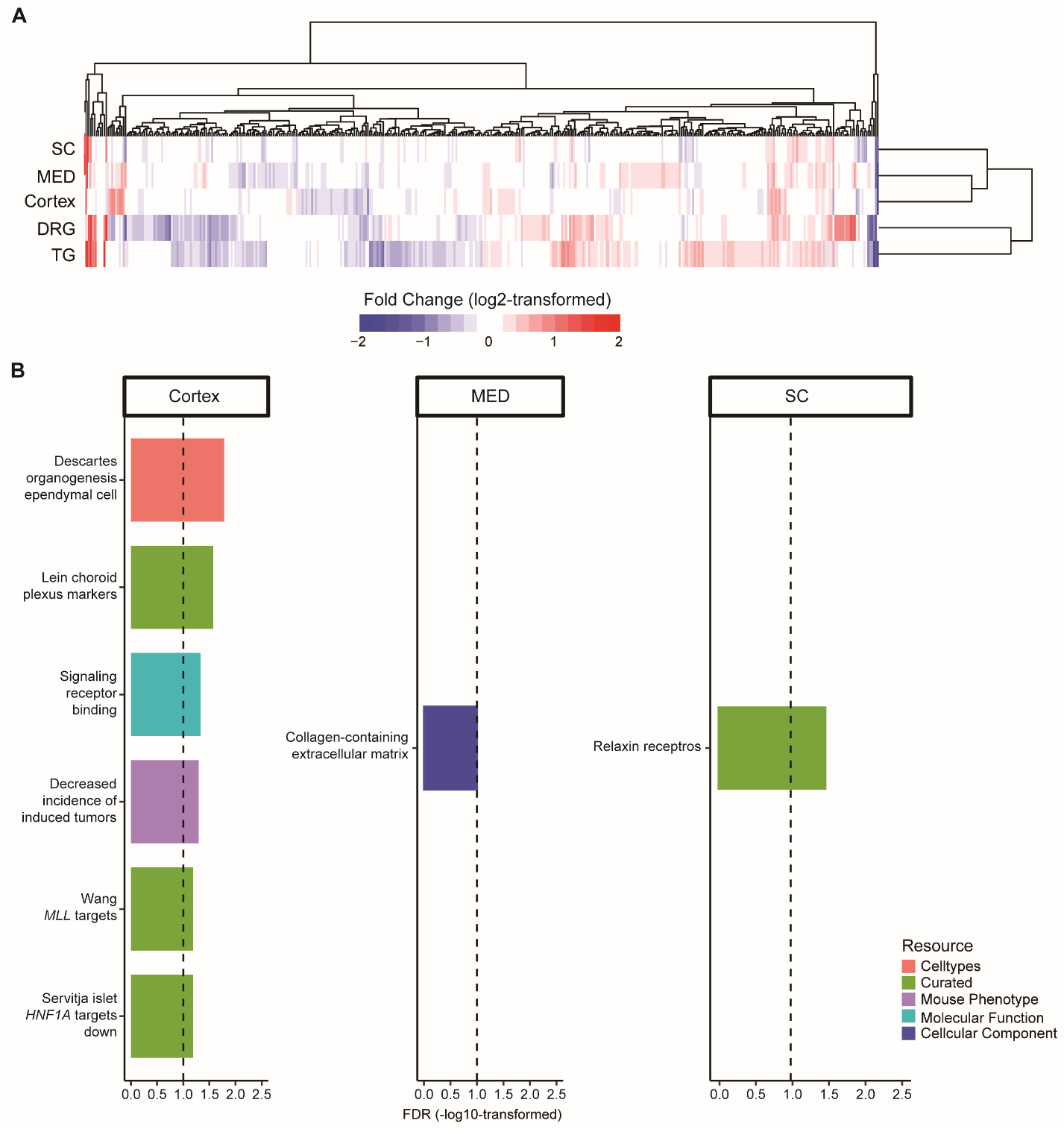


**Supplementary Figure S2. Tissue-specific DEGs and their functional enrichment.**

**(A)** The heatmap represents all the DEGs across five tissues. Each column represents a gene, and each row represents a tissue. The red color domain represents upregulation between FD and Control while the blue color domain represents downregulation, where the expression changes are measured by log2-transformed fold changes. The deeper the color, the stronger the fold change. **(B)** The bar plot represents the functional enrichment based on DEGs in Cortex, MED, and SC, respectively. Note, all significant terms (FDR < 0.1) are displayed per tissue. The x-axis represents the enrichment significance in -log10-transformed FDR while the y-axis represents the terms. The bar colors indicate the resources of Gene Set Enrichment Analysis (GSEA) v. MS1. The vertical black dashed line represents an FDR of 0.1.

**
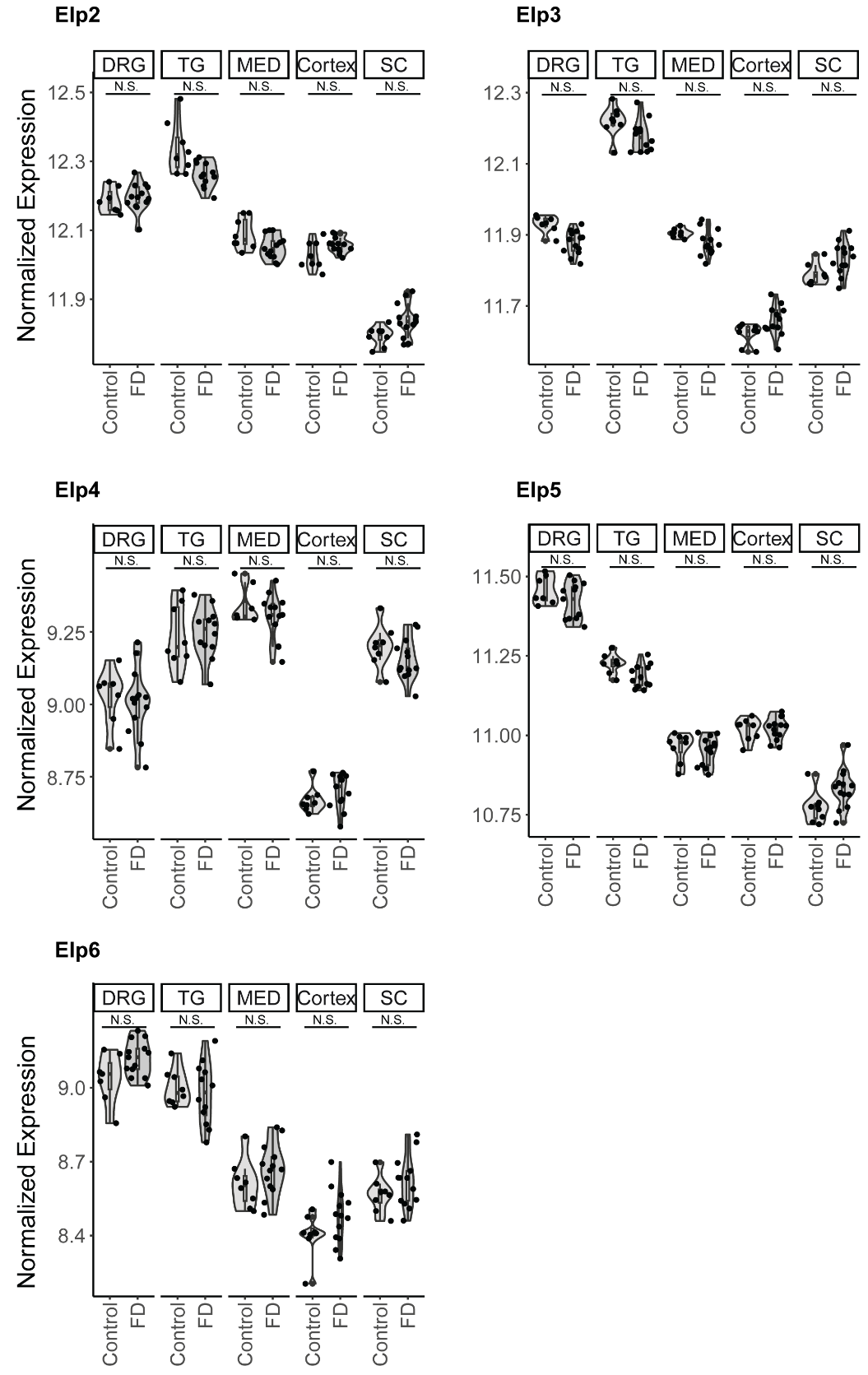
**

**Supplementary Figure S3. Gene expression comparisons of other elongator component genes between FD and Control.**

The x-axis represents the two genotypes (i.e., FD and Control) for each of the five tissues, while the y-axis represents normalized gene expression. The plot illustrates the distribution of gene expression, with each dot representing a sample. The label 'N.S.' indicates non-significance in the expression difference between the two genotypes, as determined by the Wald test.

**
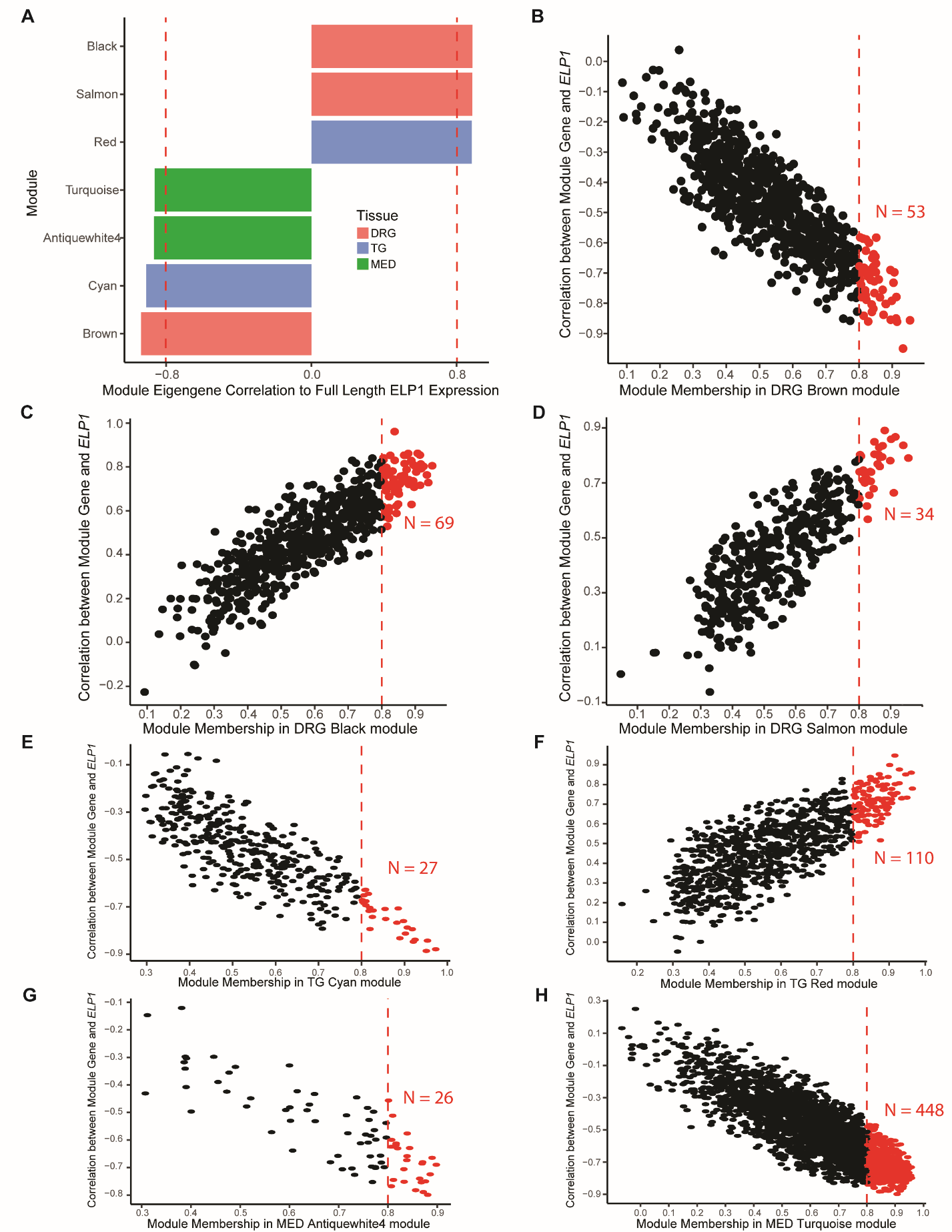
**

**Supplementary Figure S4. Identification of *ELP1* dose-responsive genes.**

**(A)** The bar plot represents how strong the full-length *ELP1* expression is correlated with the eigengene of each tissue-specific co-expression module. Only modules with an absolute Pearson correlation coefficient no less than 0.8 are displayed. The x-axis demonstrates the Pearson correlation coefficient between the full-length *ELP1* expression and an eigengene. The y-axis indicates the module names. The bars are colored by tissue types. The vertical dashed red line indicates Pearson correlation coefficients at -0.8 and 0.8, respectively. **(B-H)** The scatter plots represent the relationships among each gene in the modules in panel **(A)**, module eigengenes, and the full-length *ELP1* expression. The x-axis demonstrates the module membership, which is the Pearson correlation coefficient between each gene in the module and the module’s eigengene. The y-axis demonstrates the Pearson correlation coefficient between each gene in the module and the full-length *ELP1* expression. The vertical dashed red line indicates a Pearson correlation coefficient cutoff at 0.8. The genes to the right side of this cutoff line are defined as *ELP1* dose-responsive genes and displayed in red dots. **(B)** The co-expression module of DRG Brown. **(C)** The co-expression module of DRG Black. **(D)** The co-expression module of DRG Salmon. **(E)** The co-expression module of TG Cyan. **(F)** The co-expression module of TG Red. **(G)** The co-expression module of MED Antiquewhite4. **(H)** The co-expression module of MED Turquoise.

**
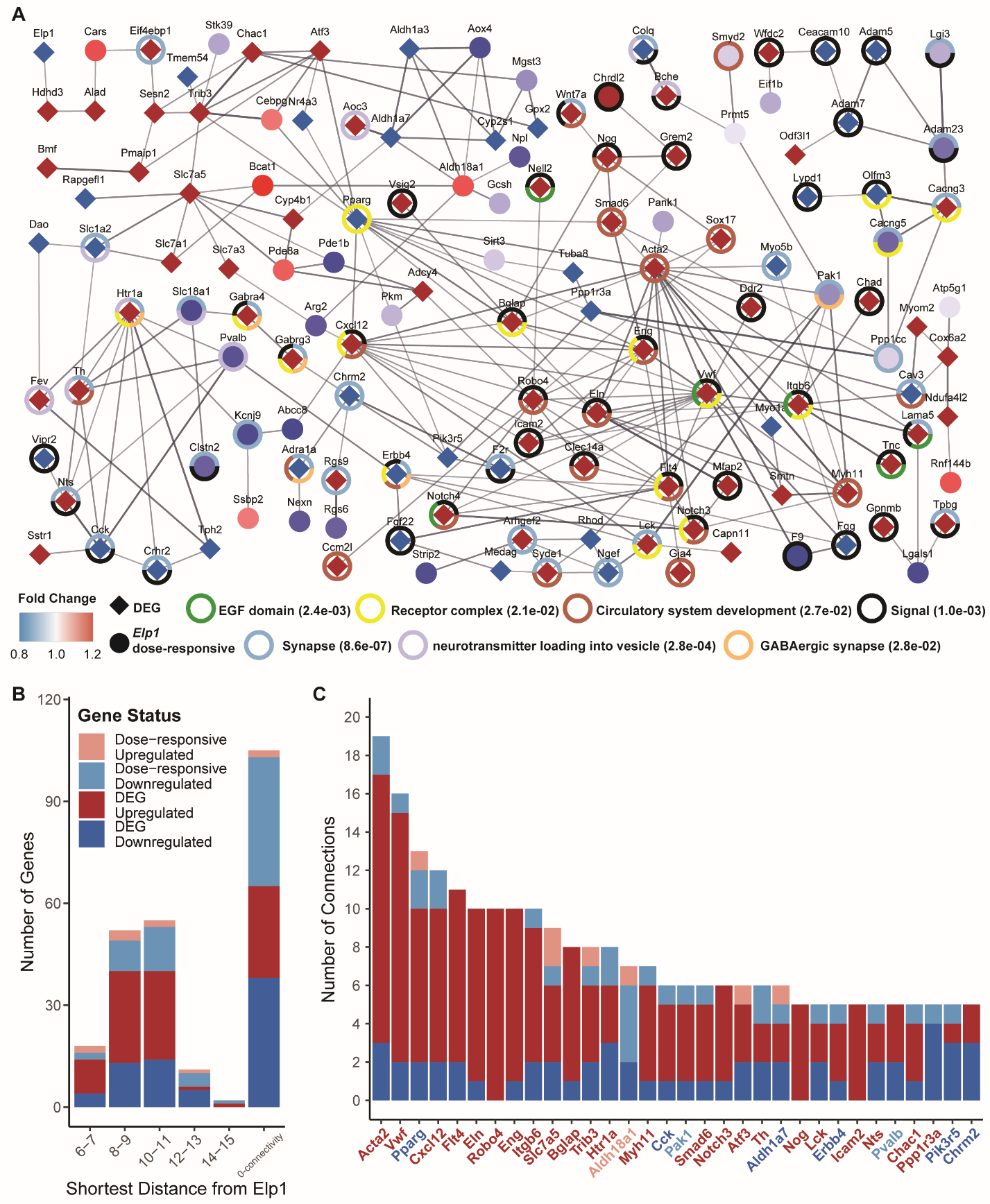
**

**Supplementary Figure S5. TG-specific dysregulated gene network due to ELP1 reduction.**

**(A)** The dysregulated gene network in TG. Each node is either a DEG indicated by a diamond shape or an ELP1 dose-responsive gene indicated by a round shape. The colors for the nodes reflect the fold changes in the genes between FD and Control. The red color domain represents upregulation between FD and Control while the blue color domain represents downregulation. The deeper the color, the stronger the fold changes. Each edge represents a potential interaction between the two connected genes, where only an interaction score of more than 0.4 (default) in String-DB is displayed. The thicker the edge, the higher the interaction score. Only the dysregulated genes with at least one interaction are displayed. The rings outside the nodes represent significant functional enrichment with FDR < 0.1 using all the dysregulated genes (i.e., DEGs and ELP1 dose-responsive genes). The associated functional enrichment terms with the ring colors are given, where the values in the brackets are the enrichment FDR values for the terms. **(B)** The bar plot demonstrates the number of dysregulated genes in TG at different distances to ELP1. The x-axis represents the distance of the shortest path to a gene. The genes in the “0-connectivity” distance category refer to those dysregulated genes not displayed in panel **(A)** due to an interaction score >= 0.4. The y-axis represents the number of genes at each distance. **(C)** The bar plot demonstrates hub genes in TG ranked by their number of connections to the neighbor genes in the network of panel **(A)**. The x-axis represents the hub gene names, where each gene is colored according to its dysregulation direction and gene category.

**
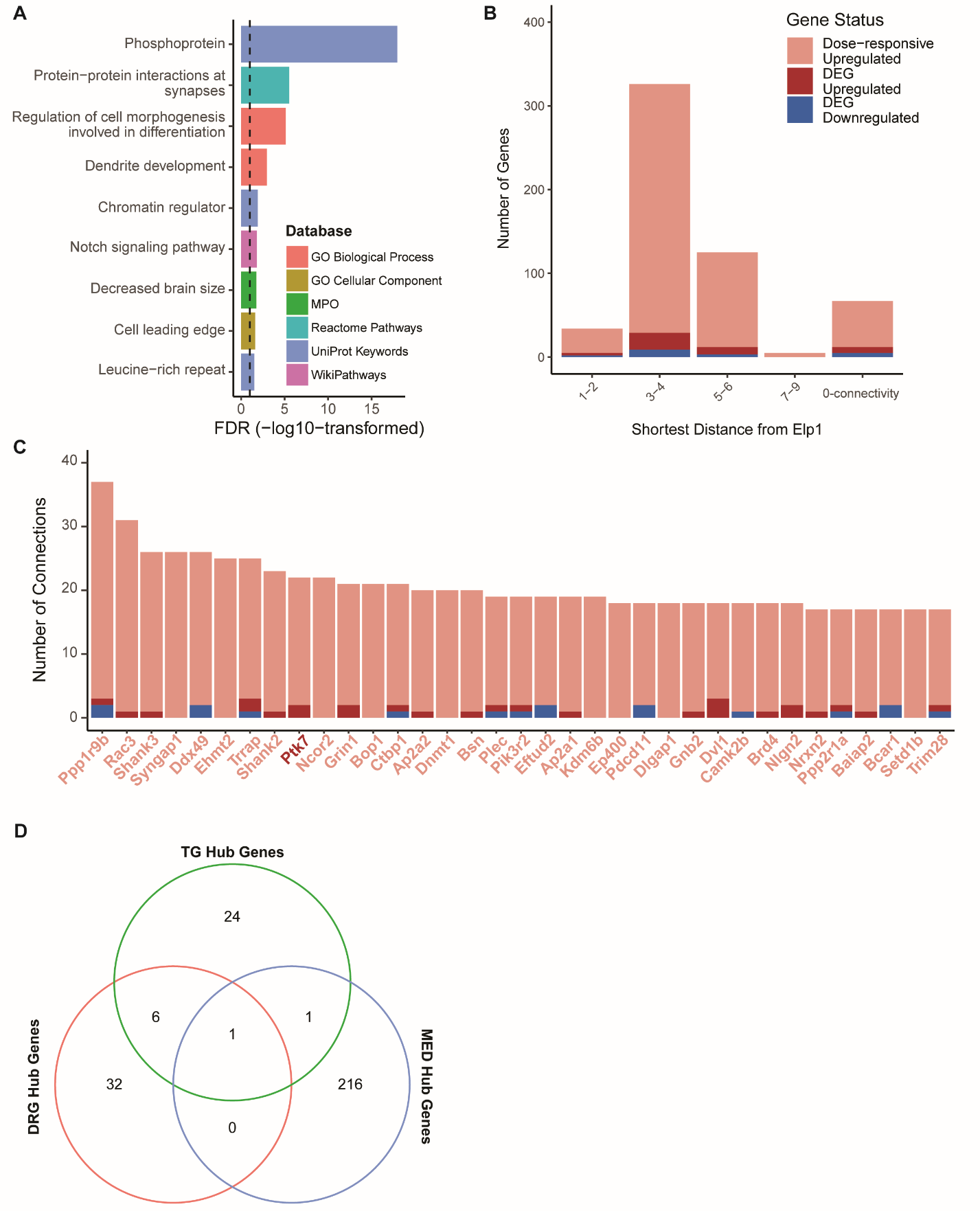
**

**Supplementary Figure S6. MED-specific dysregulated gene network due to ELP1 reduction.**

**(A)** The bar plot represents the GO functional enrichment term with FDR < 0.1 using all the dysregulated genes (i.e., DEGs and ELP1 dose-responsive genes). The x-axis demonstrates the FDR value of enrichment in the -log10-transformed scale. The y-axis demonstrates the GO terms where bars are colored by GO resources. The vertical dashed black line indicates an FDR of 0.1. **(B)** The bar plot demonstrates the number of dysregulated genes in MED at different distances to ELP1. The x-axis represents the distance of the shortest path to a gene. The genes in the “0-connectivity” distance category refer to those dysregulated genes without any connected path to ELP1. The y-axis represents the number of genes at each distance. **(C)** The bar plot demonstrates hub genes in MED ranked by their number of connections to the neighbor genes in the network of panel **(A)**. The x-axis represents the hub gene names, where each gene is colored according to its dysregulation direction and gene category. **(D)** The Venn diagram shows the overlaps among the hub genes of the dysregulated gene networks in DRG, TG, and MED. The numbers represent the amount of overlapped genes.

**
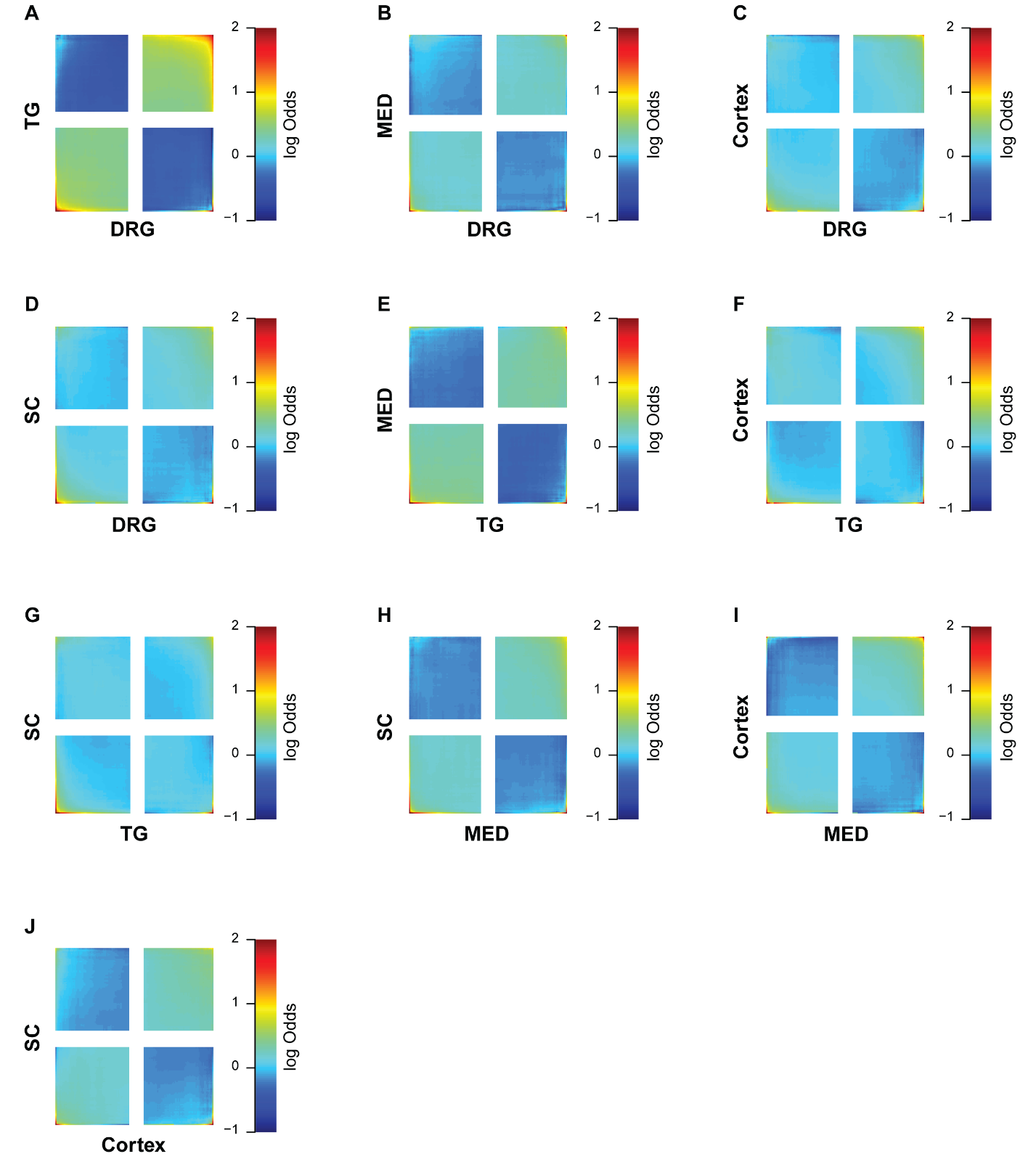
**

**Supplementary Figure S7. Concordance of FD transcriptomic changes between any two tissues .**

**(A-J)** Each plot demonstrates the concordance of expression changes for shared genes between two tissues. The x-axis represents the rank of these genes’ degree of differential expression (DDE, calculated as the sign of log2-fold change times -log10-transformation of FDR between FD and Control) from the largest (left) to the smallest (right). The y-axis represents the rank of these genes’ DDE from the largest (bottom) to the smallest (top). Each pixel represents an overlap of two sets of genes: one set consists of genes from Rank 1 to the Rank of this pixel’s y-axis value in the y-axis tissue while the other set consists of genes from Rank 1 to the Rank of this pixel’s x-axis value in the x-axis tissue. The pixel’s color represents the significance of the overlap between the two sets of genes, calculated by odds ratio (log-transformed) from the Chi-squared test. These overlaps take into account directionalities of expression changes and can be divided into four quadrants, which represent upregulation in both tissues (*bottom-left*), downregulation in both tissues (*top-right*), upregulation in x-axis tissue but downregulation in y-axis tissue (*top-left*), and downregulation in x-axis tissue but upregulation in y-axis tissue (*bottom-right*). The comparisons are between **(A)** DRG and TG, **(B)** DRG and MED, **(C)** DRG and Cortex, **(D)** DRG and SC, **(E)** TG and MED, **(F)** TG and Cortex, **(G)** TG and SC, **(H)** MED and SC, **(I)** MED and Cortex, and **(J)** Cortex and SC.

**
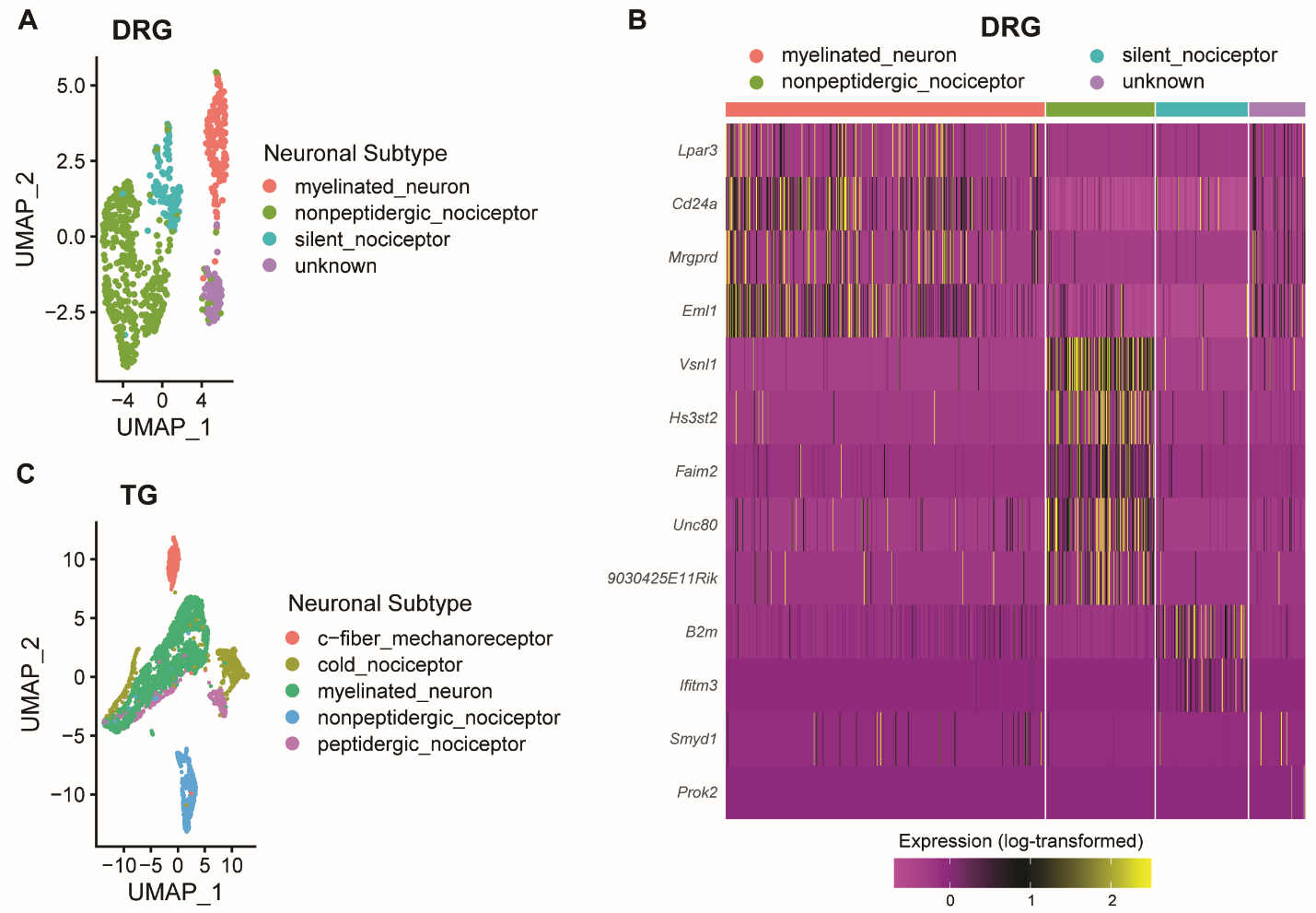
**

**Supplementary Figure S8. Re-analyses of publicly available scRNA datasets of mouse DRG and TG.**

**(A)** The scatter plot represents the 2D UMAP space of mouse DRG. Each dot represents a cell and is colored according to its assigned neuronal subtype from the re-analysis. **(B)** The heatmap represents the expression of selected markers of each neuronal subtype in mouse DRG. The rows represent neuronal subtype markers while the columns represent cells grouped by their assigned neuronal subtypes from the re-analysis. The gene expression is measured in the log-transformed scale and colored. The purple domain reflects lower expression while the yellow domain represents higher expression. **(C)** The scatter plot represents the 2D UMAP space of mouse TG. Each dot represents a cell and is colored according to its assigned neuronal subtype from the re-analysis.
